# Supplementary material for: Serological and Uterine Biomarkers for Detecting Endometritis in Mares
Source: Animals (Basel). 2023 Jan 11;13(2):253. doi: 10.3390/ani13020253 (PMC9854937; doi:10.3390/ani13020253)
Supplement: Supplementary file 1 [file animals-13-00253-s001.zip › animals-2106950-supplementary.pdf]

Supplement File S1.

Additional information on the methods of biochemical analyses used in this study

FRAP

Dose–response of iron (II) sulphate heptahydrate applied as standard

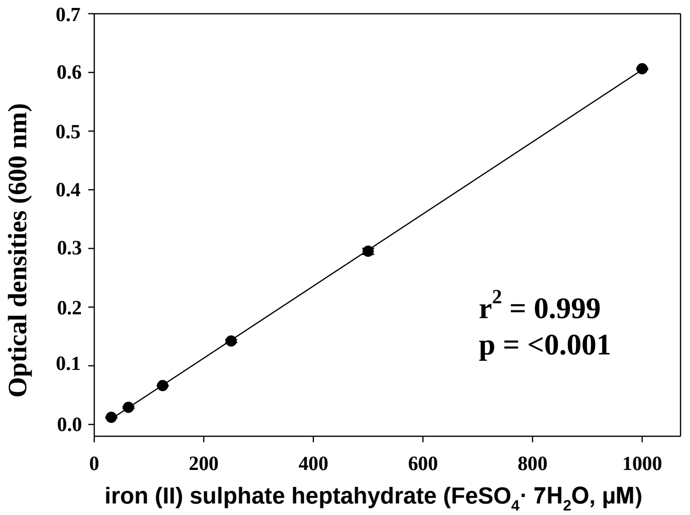

The regression line has a slope=0.0006144 and an intercept=−0.0099303. The optical densities (ODs) of the reaction mixture were read at 600 nm with a microplate reader (Model 550, BioRad, Segrate, Milan, Italy).

TAC

Dose–response of ascorbic acid applied as standard

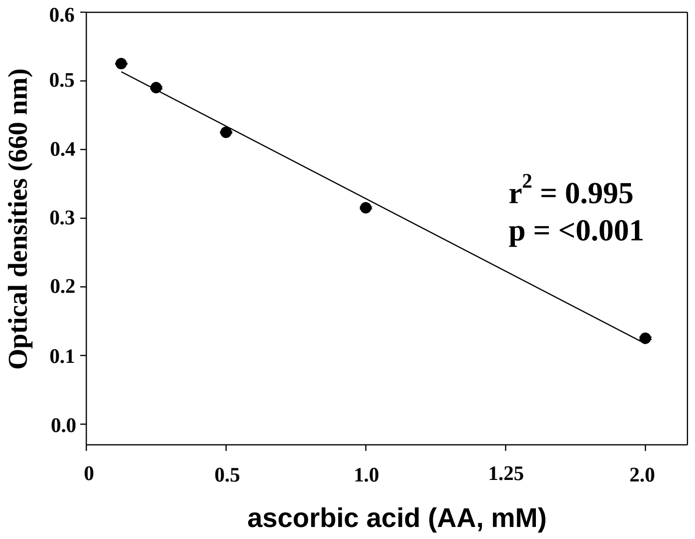

The regression line has a slope=−0,2113441 and an intercept=0.5397917. The optical densities (ODs) of the reaction mixture were read at 660 nm with a microplate reader (Model 550, BioRad, Segrate, Milan, Italy).

FRSA

FRSA of each sample was compared with a 1 mg mL^{-1} ascorbic acid (AA) solution and the data were expressed as AA equivalents applying the following equation:
AA equivalents (mg mL^{-1}) = (OD sample/OD AA)
where: OD sample is the absorbance of the sample, OD AA is the absorbance of the AA solution.
The ODs of the reaction mixture were read at 520 nm with a microplate reader (Model 550, BioRad, Segrate, Milan, Italy).

TTL

TTL of samples are presented in terms of μM and the analytical data are obtained as difference between a first and a second absorbance read at 412 nm with a spectrophotometer (SmartSpec 3000 UV/Vis, Bio-Rad,Segrate, Italy), after adding DTNB in absolute methanol. The concentration was calculated applying the following equation:
TTL (μM) = (OD_2 – OD_1) x [(1.07/0.05)/13.6] x 1000
Where:
OD_1 is the first absorbance value of sample; OD_2 is the second absorbance value of sample; 1.07 is the final volume of the reaction mixture; 0.5 is the sample volume; 13.6 is the molar extinction coefficient (ε_{412nm}, mM) of DTNB.

TOS

Dose–response of *tert*-butyl hydroperoxide applied as standard

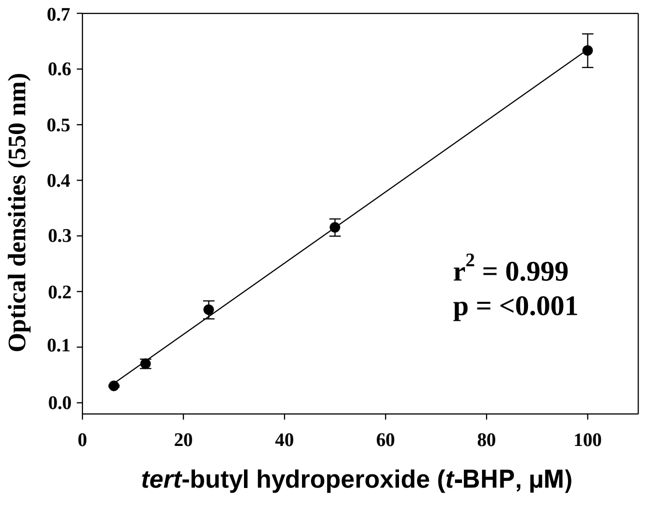

The regression line has a slope=0.0064011 and an intercept=–0.0050417. The optical densities (ODs) of the reaction mixture were read at 560 nm with a microplate reader (Model 550, BioRad, Segrate, Milan, Italy).

NO_x

Dose–response of sodium nitrate applied as standard

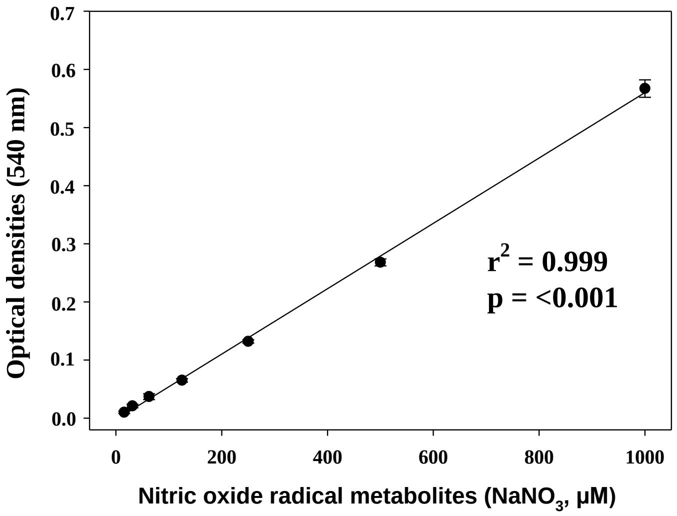

The regression line has a slope=0,0005224 and an intercept=–0.0022786. The optical densities (ODs) of the reaction mixture were read at 540 nm with a microplate reader (Model 550, BioRad, Segrate, Milan, Italy).

AOPP

Dose–response of chloramine-T applied as standard

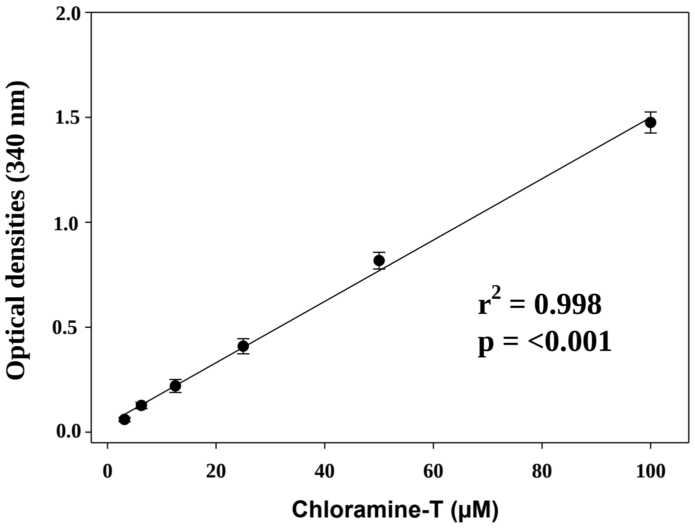

The regression line has a slope=0.0332836 an intercept=0.0146809. The optical densities (ODs) of the reaction mixture were read at 340 nm with a spectrophotometer (SmartSpec 3000 UV/Vis, Bio-Rad,Segrate, Italy).

MPO

Analytic method (in detail)
Fifteen µl of serum were diluted with 135 µl of HBSS without Ca^{+2} or Mg^{+2} in flat-bottomed 96 well plates. Then, 50 µl of TMB (Sigma) and 5 mM H_2O_2 (both substrates of MPO and prepared daily) were added. The colour-change reaction was stopped after 2 min by adding 50 µl of 4 M sulfuric acid (H_2SO_4). The optical density was read at 450 nm with a microplate reader (Model 550, BioRad, Segrate, Milan, Italy).

Protease activity

Analytic method (in detail)
The protease activity of the sample was measured as its ability to degrade azocasein. The activity of the sample was referred to the capacity of a trypsin solution (1 mg mL^{-1}) and expressed as a percentage of activity according to the following formula:
Protease activity (as trypsin activity, %) = $(OD_{sample}/OD_{trypsin})$ x 100
The optical densities (ODs) of the reaction mixture were read at 450 nm with a microplate reader (Model 550, BioRad, Segrate, Milan, Italy).

Antiprotease activity

Analytic method (in detail)
The antiprotease activity of the sample expresses its ability to oppose the protease activity of trypsin. The results are presented as percentage of inhibition of trypsin activity against the positive control, the trypsin solution, adopting the following formula:
Antiprotease activity (as trypsin inhibition, %) = $1 -[(OD_{trypsin}/OD_{sample})/OD_{trypsin}]$ x 100
The optical densities (ODs) of the reaction mixture were read at 450 nm with a microplate reader (Model 550, BioRad, Segrate, Milan, Italy).
